# Biosynthesis of aromatic polyketides in microorganisms using type II polyketide synthases

**DOI:** 10.1186/s12934-020-01367-4

**Published:** 2020-05-24

**Authors:** Jia Wang, Ruihua Zhang, Xin Chen, Xinxiao Sun, Yajun Yan, Xiaolin Shen, Qipeng Yuan

**Affiliations:** 1 State Key Laboratory of Chemical Resource Engineering, Beijing University of Chemical Technology, 15 Beisanhuan East Road, Chaoyang District, Beijing, 100029 China; 2 School of Chemical, Materials and Biomedical Engineering, College of Engineering, University of Georgia, Athens, GA 30602 USA

**Keywords:** Aromatic polyketides, Type II polyketide synthases, Starter units, Chain length, Tailoring reactions

## Abstract

Aromatic polyketides have attractive biological activities and pharmacological properties. Different from other polyketides, aromatic polyketides are characterized by their polycyclic aromatic structure. The biosynthesis of aromatic polyketides is usually accomplished by the type II polyketide synthases (PKSs), which produce highly diverse polyketide chains by sequential condensation of the starter units with extender units, followed by reduction, cyclization, aromatization and tailoring reactions. Recently, significant progress has been made in characterization and engineering of type II PKSs to produce novel products and improve product titers. In this review, we briefly summarize the architectural organizations and genetic contributions of PKS genes to provide insight into the biosynthetic process. We then review the most recent progress in engineered biosynthesis of aromatic polyketides, with emphasis on generating novel molecular structures. We also discuss the current challenges and future perspectives in the rational engineering of type II PKSs for large scale production of aromatic polyketides.

## Background

**Fig. 1:**
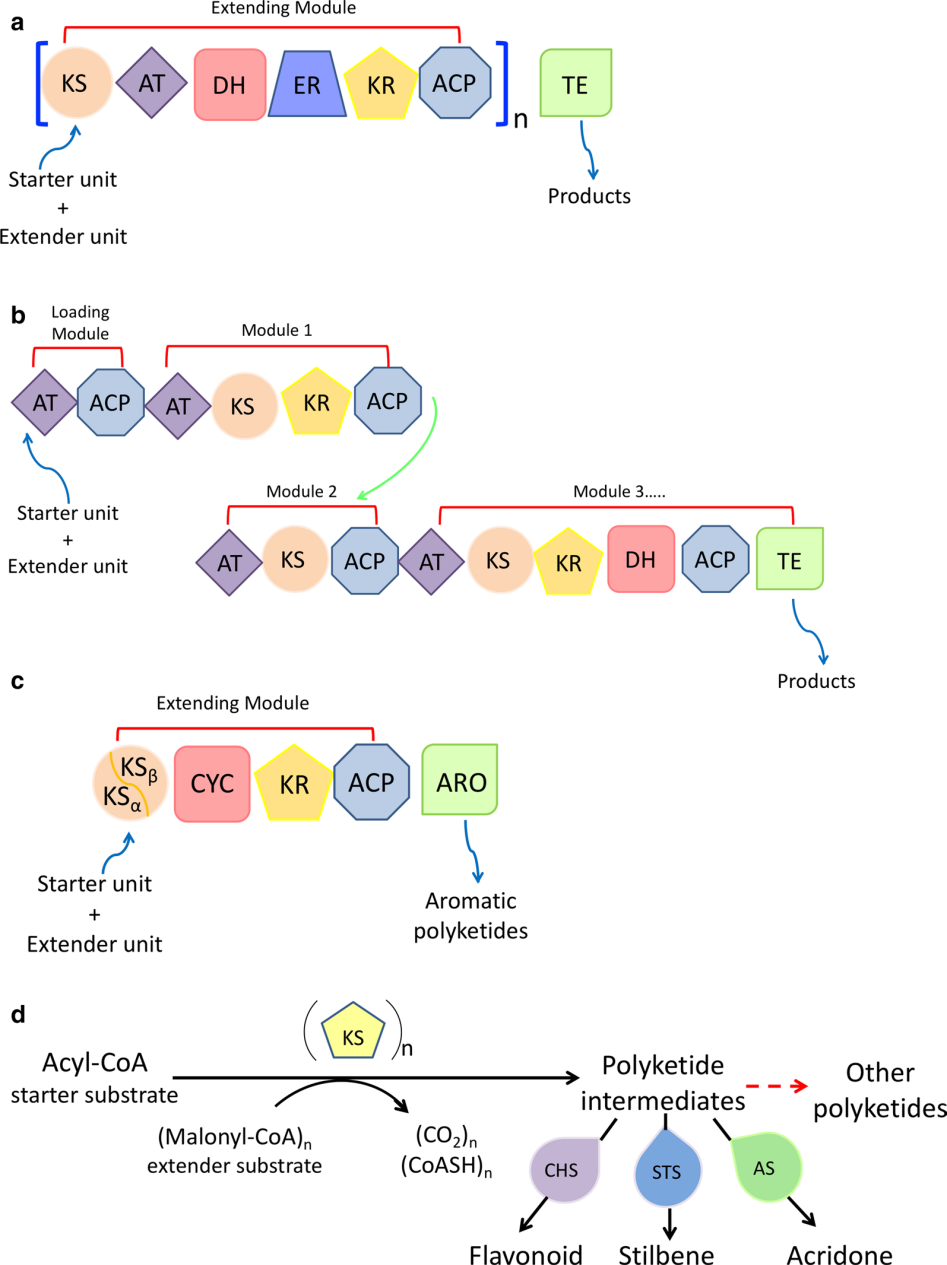
Catalytic reaction of PKSs. **a** Iterative type I PKSs; **b** Modular type I PKSs; **c** Type II PKSs; **d** Typical type III PKSs use acyl-CoA as the starter unit and malonyl-CoA as the extender unit. *KS* ketoacyl synthase, *AT* acyltransferase, *DH* dehydratase, *ER* enoylreductase, *KR* ketoreductase, *ACP* acyl carrier protein, *TE* thioesterase, *CYC* cyclase, *ARO* aromatase, *CHS* chalcone synthase, *STS* stilbene synthase, *AS* acridone synthase

Polyketides are structurally diverse and biologically active secondary metabolites derived from natural sources such as animals, plants, fungi and bacteria [1]. These compounds are widely used as clinical medicines for the treatment of various acute and chronic diseases [2]. Examples include antibacterial (erythromycin and tetracycline) [3], antitumor (anthracycline and doxorubicin) [4], antifungal (amphotericin and griseofulvin) [5], antiparasitic (avermectin) [6] and anti-cholesterol (lovastatin) [7] drugs. Polyketides are synthesized by polyketide synthases (PKSs) which are multi-domain enzymes or enzyme complexes [8], consisting of acyltransferase (AT), ketosynthase (KS), thioesterase (TE) and optional domains. The biosynthesis of polyketides is initiated by loading the starter unit acyl-Coenzyme A (CoA) on the acyl carrier protein (ACP) catalyzed by the AT domain [2]. The KS domain subsequentially elongates the carbon chain by decarboxylative Claisen condensation. The *β*-keto group can be further modified to generate different polyketide structures by additional domains, including ketoreductase (KR), dehydratase (DH) and enoylreductase (ER) domains. Finally, the TE domain hydrolyzes or cyclizes the completed polyketide chain from the ACP-domain to terminate the elongation [9]. Although sharing a similar synthetic process, PKSs can be classified into three types, type I, II, and III PKSs (Fig. 1) [10, 11]. Type I PKSs are multifunctional peptides containing linearly arranged and covalently fused domains. The type I PKSs can be further classified into iterative type I PKSs (iPKSs) (Fig. 1a) and modular type I PKSs (mPKSs) (Fig. 1b) [12]. iPKSs are mainly found in fungi and the domains are used repeatedly to catalyze multiple rounds of elongation [13, 14]. While, mPKSs are primarily found in bacteria and function in an assembly line similar to the nonribosomal peptide synthases [15]. mPKSs consist of several domains with defined functions that are separated by short spacer regions. The distinct domains work cooperatively and non-iteratively to catalyze the carbon chain elongation and functional group regeneration [16]. Type II PKSs are multi-enzyme complexes composed of monofunctional proteins (Fig. 1c). They are found predominantly in bacteria and produce diverse aromatic polyketides [17]. Different from both type I and type II PKSs, type III PKSs are mainly found in plants as simple homodimers that use CoA rather than ACP as an anchor for chain extension (Fig. 1d) [18], while both type II and type III PKSs are iterative.

Type II PKSs are mainly responsible for producing aromatic polyketides by catalyzing iterative Claisen condensation reaction usually using acetate as the starter unit [19]. Aromatic polyketides are polycyclic compounds harboring at least one aromatic ring [17]. They are an important type of natural products with antibacterial, anticancer and antiviral bioactivities, with representative examples being the above-mentioned drugs tetracyclines and anthracyclines [3, 4]. The clinical and environmental potential of aromatic polyketides has attracted increasing attention from researchers to conduct studies on the biosynthesis of these polyketides. The production process of aromatic polyketides includes the following reactions. (1) *α*-Carboxylated precursor loading: acetate is loaded onto ACP, forming acyl-ACP. (2) Iterative chain extension and ketone reduction: acyl-ACP is transferred to the KS subunit and iteratively elongated with the extender unit malonyl-CoA to form the poly-*β*-keto chain. (3) Cyclization and/or aromatization: the poly-*β*-keto chain is catalyzed by aromatases (ARO) and oxygenases to generate the aromatic polyketide core. (4) Post-modification: oxygenases, methyltransferases and glycosyltransferases catalyze this aromatic polyketide core to generate the final product [17, 20]. The characteristic of aromatic polyketides biosynthesis is the employment of a set of iteratively used enzymes. This set of enzymes is called minimal type II PKS, which contains KS_α_, chain length factors (CLF or KS_β_) and ACP subunits. The sequences of KS_α_ and CLF components are highly similar, except for a cysteine-containing active site in KS_α_ that is essential for assembling aromatic polyketides [17, 21]. A minimal PKS can solely produce some aromatic polyketides, for instance, SEK4, SEK 4b and dehydro-SEK4b [22].

Although type II PKSs and aromatic polyketides are a tribute to our life, there have been only a few articles focusing on the understanding and engineering of type II PKSs probably due to the complicated catalytic mechanisms. In contrast, type I and type III PKSs have been well-characterized and engineered to produce various bioactive compounds [18, 23, 24]. Many excellent reviews have summarized the research progress of these PKSs [18, 25–29]. With the development of biotechnology and emerged characterization of type II PKSs, great progress has been made in recent years to optimize type II PKSs for high-level production of aromatic polyketides. Meanwhile, with the rising issue of antibiotic and anticancer drug resistance, developing of diverse polyketides with new bioactivity is of particular interest. In this paper, we will briefly review the recent efforts on improving the production and broadening the spectrum of aromatic polyketides.

### Production of aromatic polyketides by employing different starter units

**Fig. 2:**
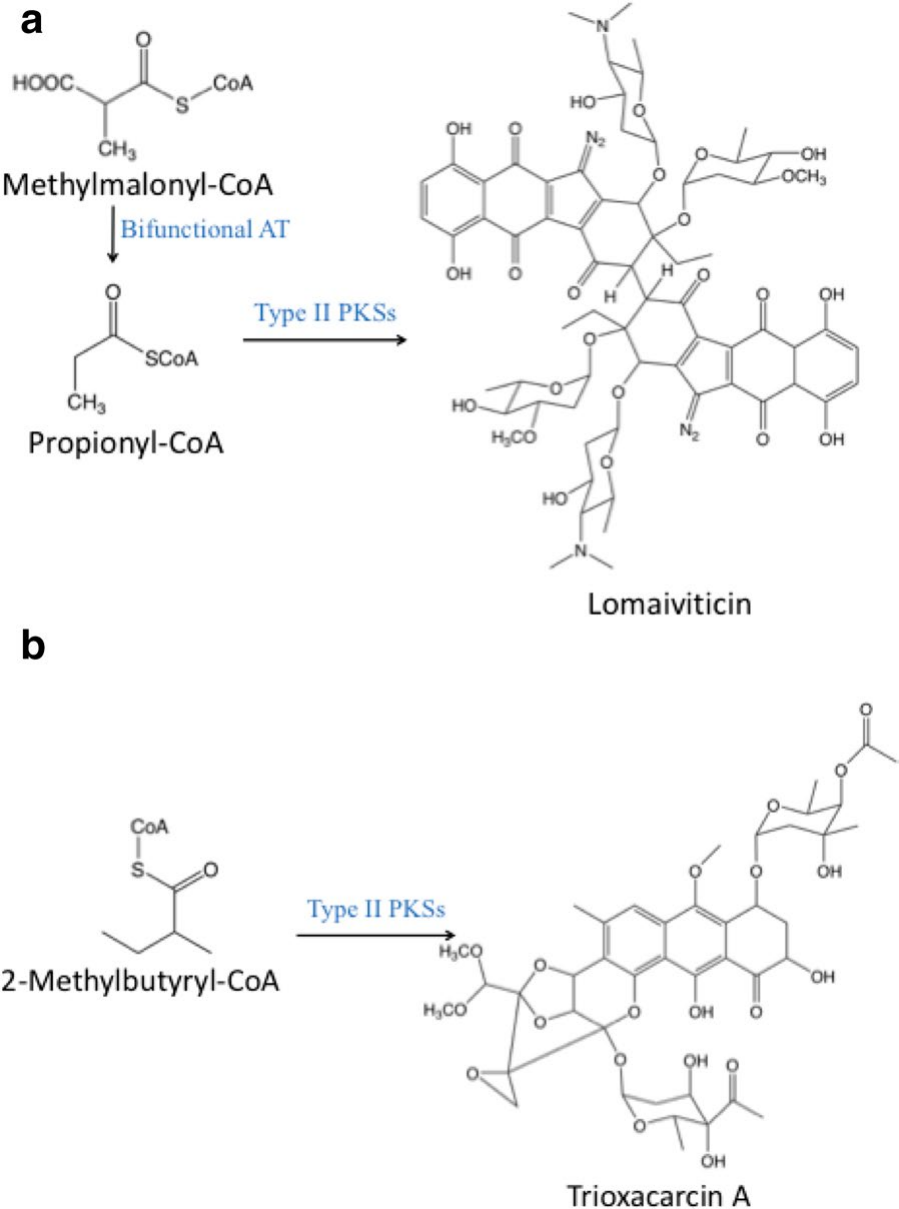
Biosynthesis of lomaiviticin and trioxacarcin A using different starter units. Type II PKSs can catalyze different starter units to synthesize different products

The first step of aromatic polyketide biosynthesis is loading of a starter unit, mostly being acetate, onto ACP. A typical case is the biosynthesis of kinamycins, a group of bacterial polyketide secondary metabolites containing a diazo group [30], which begins with the condensation of 1 acetyl-CoA and 9 malonyl-CoA molecules to form the intermediate dehydrorabelomycin [31]. While only a few type II PKSs have been identified to use other starter units, the employment of different starter units could provide additional structurally diverse products. Propionyl-CoA has been reported to be the starter unit for several aromatic polyketides. For instance, lomaiviticins, an important class of diazo-containing aromatic polyketides, have gained great interest because of their antibiotic and antitumor activities [32]. Waldman and his colleagues identified a gene cluster responsible for the biosynthesis of lomaiviticin in *Salinispora pacifica* DPJ-0019. This cluster contains a bifunctional enzyme (encoded by *Lom62*) that has both acyltransferation and decarboxylation activities and can catalyze the conversion of methylmalonyl-CoA to propionyl-CoA, which serves as a starter unit for lomaiviticin production (Fig. 2a) [19]. In addition, Otten et al. identified a gene cluster *dnr* encoding type II PKS which can also employ propionyl-CoA as the starter unit for the production of daunorubicin and doxorubicin, two famous anthracycline topoisomerase inhibitors, in *Streptomyces* spp. [33]. However, expression of this cluster was found restrained by the lack of *bldA*-tRNA in *S. peucetius* to read a rare TTA codon in *dnrO*, which is a transcriptional activator involved in the biosynthesis of daunorubicin. For purpose of activating daunorubicin biosynthesis, *bldA*-tRNA was heterologously over-expressed in *S. peucetius*. The productivity of daunorubicin was increased by 45.7% compared to that of wild type *S. peucetius* strain [34]. To obtain a more efficient doxorubicin producer, Wang et al. employed UV and ARTP (atmospheric and room temperature plasma) approaches to mutate doxorubicin producer strains. With the aid of fermentation condition optimization, the titer of doxorubicin dramatically increased from 119 mg/L to 1.1 g/L in a 5-L fermenter, being the highest reported so far [35]. Those researches offered a new starter unit for the generation of aromatic polyketides and represented the potential of large-scale production of daunorubicin.

In addition to propionyl-CoA, malonamate (3-amino-3-oxopropanoate) has been reported as the starter unit for the biosynthesis of oxytetracycline, a broad-spectrum antibiotic belonging to the tetracycline family [21, 36]. Malonamate is converted from malonyl-CoA by aminotransferase OxyD and thiolase OxyP. Then, oxytetracycline was produced after a series of reactions catalyzed by 19 enzymes encoded by *oxy* gene cluster, with the addition of extender units malonyl-CoA [37]. Based on these studies, heterologous production of oxytetracycline was achieved recently. Stevens et al. successfully engineered *E. coli* to produce 2 mg/L oxytetracycline by co-expressing the biosynthesis gene cluster with an alternative sigma factor σ^54^ from the oxytetracycline native producer *S. rimosus* [38]. A fast-growing strain *S. venezuelae* WVR2006 was also chosen as the producer to accumulate oxytetracycline. The titer of oxytetracycline reached 431 mg/L within 48 h by overexpressing the *oxy* gene cluster [39]. In order to further increase the production of oxytetracycline, *S. rimosus* strain was engineered for enhanced self-resistance level by overexpression of the ribosomal protection protein and two efflux proteins, leading to a remarkable increase in titer of 7.59 g/L oxytetracycline in shake flasks [40]. By assembling oxytetracycline and chelocardin biosynthetic pathways, Lešnik et al. obtained 2-carboxamido-2-deacetyl-chelocardin (CDCHD), a new class of tetracycline that uses malonamate as the starter unit. Compared to chelocardin, the new antibiotic CDCHD has a carboxamido moiety and showed higher in vitro activities against clinical isolates [41].

In addition to propionyl-CoA and malonamate, several other starter units have been employed to produce novel aromatic polyketides. Recently, 2-methylbutyryl-CoA was found to initiate the scaffold trioxacarcins biosynthesis by Zhang et al. via ^13^C-labeling experiments (Fig. 2b) [42]. This research provides an opportunity to get more potential drugs by identifying a possible new starter unit. A further study showed that *p*-nitrobenzoic acid was synthesized from the native intermediate *p*-aminobenzoic acid catalyzed by a non-heme di-iron monooxygenase AurF [43]. Additionally, the structural diversity of polyketides can also be expanded by utilizing modified amino acids as the starter units. 4,5-Dichloropyrrolyl-*S*-carrier protein was found to be the starter unit of pyoluteorin biosynthetic in *Pseudomonas fluorescens* Pf-5 [44]. The discovery of more distinct starter units and their corresponding native producers can facilitate the production of various aromatic polyketides.

### Production of aromatic polyketides by varying chain length

In addition to altering starter units, changing the chain length can also yield a variety of polyketides. Different from type I and type III PKSs, type II PKSs are aggregates of mono-functional proteins, containing a KS_α_-CLF complex that can determine the carbon chain length of aromatic polyketides by controlling the number of Claisen condensations [45]. Previous study showed that *act* KS_α_-CLF or *tcm* KS_α_-CLF act as the chain length determination subunit for the synthesis of C-16 or C-20 chain length polyketides, respectively [1]. In order to further understand the mechanism of structural determination, both *act* KS_α_-CLF and *tcm* KS_α_-CLF were separated into 12 cassettes (6 KS_α_ and 6 CLF parts). KS_α_-CLF hybrids were then constructed by single replacements with mutual counterparts based on sequence homology. The results showed that cassette D in KS_α_, cassette C and D in CLF are the primary structural determinants for carbon chain length [46].

To date, type II PKSs naturally synthesizing aromatic polyketides with chain length of C-10 to C-30 have been reported [17, 47]. To our surprise, an increasing number of new aromatic polyketides with different carbon chain lengths have been continuously explored in recent years. One C-13 and two new C-18 aromatic polyketides huanglongmycin, HLM A, HLM B and HLM C, were identified from *Streptomyces* sp. CB09001 which was isolated from Xiangxi, China. HLM A-C showed broad antibacterial spectrum and HLM A also exhibited antitumor activity with moderate cytotoxicity [48]. Saccharothrixones A–D are synthesized by *Saccharothrix* sp. 10-10. The molecular formula of saccharothrixones A is C_23_H_22_O_11_ and the other three compounds are C-24 polyketides. Remarkably, saccharothrixone D displayed in vitro antitumor activity [49]. These studies enlarged the spectrum of aromatic polyketides and offered new potential drugs. In some cases, KS_α_-CLF contributes to longer chains formation for production of new aromatic polyketieds. SanFG is the dedicated KS_α_-CLF to generate A-74528, a C-30 aromatic polyketide. A-74528 is an antiviral drug owing to its inhibitory effect on the regulatory enzyme 2′,5′-oligoadenylate phosphodiesterase (2′-PDE) [50]. The gene cluster in *Streptomyces sp.* SANK 61196 responsible for the biosynthesis of A-74528 was identified and sequenced. Gene *samF* and *samG* encode KS_α_ and CLF, respectively. The introduction of this gene cluster into *S. lividans* K4-114 led to only trace amount of A-74528 (less than 1 mg/L) [51]. This titer was further increased to 3 mg/L by identifying the essential pathway genes (*sanFGH*, *sanI*, *sanJ* and *sanS2C*) and refactoring the minimal biosynthetic pathway in the host strain *S. coelicolor* CH999 [52]. Although the titer of A-74528 is still low, those efforts offer an opportunity for further studying on enhancement of its titer.

The variation of actinorhodin chain length has been studied by engineering its KS_α_-CLF complex. Actinorhodin, a C-16 polyketide with multiple bioactivities, is the most-characterized type II PKSs product found in *S. coelicolor* A3 [53]. The gene cluster *act* is responsible for the synthesis of actinorhodin and *act* KS_α_-CLF contributes to the chain length determination [53, 54]. On this basis, a set of *act* KS_α_-CLF mutants were constructed to change amino acid (residues 109, 112, and 116) residue size by employing site-directed mutagenesis. The results showed a smaller size of those amino acid residues in *act* CLF led to longer reaction channel and made two additional of Claisen condensations possible. New aromatic polyketides with the carbon chain length of C-20 and C-24 were produced by the host strain carrying the obtained *act* CLF mutants [55]. This research provided a new direction for the rational design of CLF subunit to alter the chain length of aromatic polyketides. In recent years, some efforts were focused on developing strategies of improvement the production of actinorhodin. For example, the TraA-like protein ZouA and its recombination sites RsA and RsB that are essential for the amplification of *kan* gene cluster in *S. kanamyceticus*, were inserted into *S. coelicolor* genome flanking *act* gene cluster. The strains with increased copy numbers of *act* gene cluster were selected by stress-based screening and resulted in 20-fold-increase of actinorhodin yield [56]. An xdhR-encoded inhibitor, which negatively regulates actinorhodin biosynthesis through binding the intergenic region of xdhABC, were deleted for enhanced production of actinorhodin by 2.5 fold [57]. Lysoquinone-TH1 has antibacterial activity as well as pharmaceutical effects towards asthma (by inhibiing the target protein, phosphodiesterase 4) and chronic obstructive pulmonary disease. With the expression of *llpE*, encoding for lysolipin CLF, and other minimal type II PKSs genes of lysolipin, lysoquinone-TH1 was successfully synthesized in *S. albus* after condensing 13 acetate extenders. When condensing 15 acetate extenders, lysoquinone-TH2 was produced [58]. This study provides a promising strategy for the production of non-natural analogs derived from natural compounds utilizing different numbers of extenders. In addition to engineering the KS_α_-CLF complex, other approaches to achieve variant chain length of aromatic polyketides have been explored. For instance, oxytetracycline is a C-19 aromatic polyketide, whose chain length can be altered by disrupting an anhydrotetracycline oxygenase to generate a new C-17 polyketide [59]. Recently, Du et al. reported that IgaPKS, which belonged to a new subfamily of type II PKSs, can synthesize unsaturated carboxylic acids with carbon chain lengths of C-8, C-10, C-12 and C-14 [60]. This research demonstrates a new opportunity to employ type II PKSs for the production of carboxylic acids. The discovery and characterization of aromatic polyketide synthesis complexes for different chain lengths is a promising strategy to created more novel aromatic polyketides.

### Production of aromatic polyketides by combining tailoring reactions

The complexity and diversity of aromatic polyketides with new bioactivities can be endowed by tailoring reactions. The common tailoring reactions involved in the biosynthesis of aromatic polyketides are oxidation, methylation and glycosylation. Understanding and engineering of such modification processes can help derive different new aromatic polyketides by rationally combining the tailoring reactions.

Hydroxylation, epoxidation, dehydrogenation, quinone formation and oxidative rearrangement are the major oxidative tailoring reactions. The corresponding oxygenases include flavin-dependent oxygenases, anthrone-type oxygenases and cytochrome P450-dependent monooxygenases [17, 20]. EncM, a FAD-dependent oxygenase, catalyzes oxidative Favorskii-type rearrangement reaction which is a pivotal step in enterocin biosynthesis. This enzyme has a broad substrate spectrum and can also generate 5-deoxyenterocin and wailupemycin analogues [61]. The antitumor drug mithramycin A can be converted from premithramycin B, a tetracyclic intermediate, through Baeyer–Villiger oxygenation catalyzed by a FAD-dependent oxygenase, MtmOIV [62]. To improve the production of mithramycin A, the whole gene cluster was constructed into *S. lividans* TK24 along with the deletion of interfering secondary metabolite genes. The best strain produced 3 g/L mithramycin A [63]. Seven mithramycin analogues were generated by using combinatorial biosynthesis strategy in *S. argillaceus* M7C1 [64]. One of these new compounds, EC-8042, was identified to enhance the inhibitory activity towards Ewing sarcoma and showed higher inhibition activity and lower toxicity than mithramycin A [65]. This study exhibited the promising application of aromatic polyketide derivatives.

Anthrone-type oxygenases are responsible for the oxidation of anthrone-type intermediates to the related quinones without cofactors or metal ions. Shen et al. first reported an anthrone-type monooxygenase (TcmH) that can convert naphthacenone tetracenomycin F1 into 5,12-naphthacenequinone tetracenomycin D3 [66]. ActVA-orf6 and AknX are well-characterized anthrone-type monooxygenases involved in quinone-forming reactions [67, 68]. The crystal structure of ActVA-orf6 was determined by Sciara and co-workers. Based on the structural similarity, ForS and ForU from *Lechevalieria fradiae* and *Pseudonocardiales bacteria* belonging to ABM superfamily were characterized as the monooxygenase for the biosynthesis of fasamycin [69]. This study uncovered a new role of monooxygenases in the metabolism of aromatic polyketides.

On the other hand, there have been few cytochrome P450-dependent monooxygenases identified in bacteria until now. EncR and DoxA from *S. maritimus* and *S. peucetius*, respectively, have been reported to assist in the conversion of deoxyenterocin into enterocin. To catalyze this reaction, EncR requires ferredoxin and DoxA needs NADPH as cofactors [70, 71]. Furthermore, DoxA also contributes to the biosynthesis of doxorubicin, daunorubicin as well as anthracycline and its analogues [71].

**Fig. 3:**
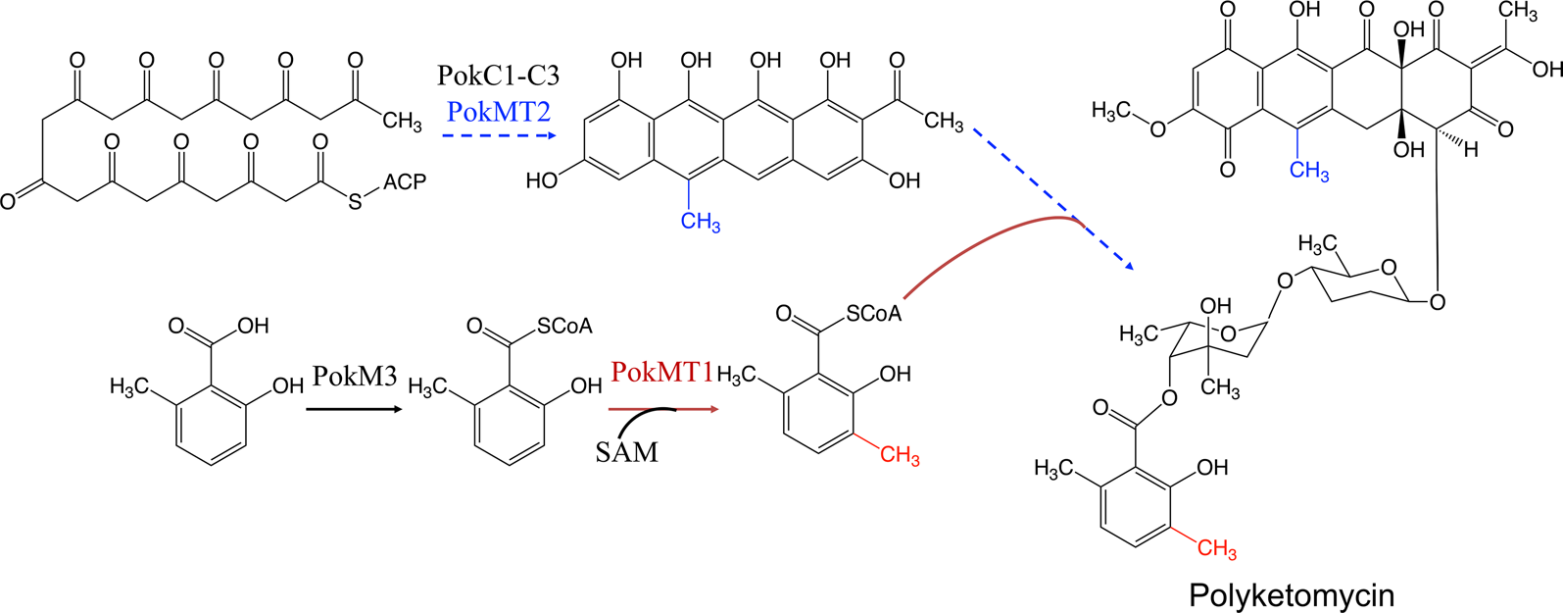
A new pathway logic for biosynthesis of polyketomycin. *SAM* S-adenosyl methionine, *PokC1-C3* cyclase, *PokMT2* C-methyltransferase, *PokM3* 6-methylsalicylic acid-AMP ligase, *PokMT1* C-methyltransferase

Methylation is another major type of tailoring reaction that aids in expanding the diversity of aromatic polyketides. The methyltransferases (MTs)-mediated reactions usually transfer a methyl group from a donor such as *S*-adenosyl-l-methionine (SAM) to the C, N or O atoms on acceptor targets. MTs are categorized into C-MTs, O-MTs and N-MTs by which of those atoms are modified. DutMT1, located in the *dut* gene cluster was identified from *S. minoensis* NRRL B-5482 by genome sequencing. To further study the function of this gene, aiming gene deletion combined with intermediates identification experiments were performed. This enzyme was then identified as a C-MT that transfers a methyl group to 12-desmethyl-dutomycin to yield dutomycin [72]. Two C-MTs were found to involve in the biosynthesis of polyketomycin (POK), an antitumor aromatic polyketide in *S. diastatochromogenes* Tü6028 (Fig. 3). PokMT2 is able to methylate the substrate at C6 position, while PokMT1 is responsible for methylation of another intermediate 6-methylsalicylyl-CoA to form 3,6-DMSA moiety of the POK molecule [73] (Fig. 3). In order to understand the reaction process of POK biosynthesis, Guo et al. characterized PokMT2 and an ATP-dependent CoA ligase, PokM3, and provided an unusual way for the generation of POK [74] (Fig. 3). This study paved the way for investigation of the catalytic mechanisms of C-MTs in polyketides biosynthesis.

Since O-MTs from microorganisms commonly have a wide substrate scope, various substrates can be introduced to expand the library of aromatic polyketides. Three O-MTs: TcmO, TcmN and TcmP were reported to participate in tetracenomycin (Tcm) biosynthesis. TcmO methylates C8 hydroxy group of Tcm D3 to generate 8-*O*-methyl-Tcm D3, which can then be modified by TcmN and TcmP individually. TcmN adds a methyl group on C3 hydroxy group of 8-*O*-methyl-Tcm D3 forming Tcm E. TcmN can also methylate Tcm D3 to generate Tcm B3. While TcmP transfers a methyl group to C9 carboxyl yielding 9-carboxymethyl-8-O-methyl-Tcm D3, respectively. Likewise, TcmP can also accept Tcm B3 as the substrate and convert it to 9-carboxymethyl-Tcm B3. The broad substrate spectrum of these O-MTs shows the potential for producing diverse Tcms [75].

DnrK is another well-studied O-MT that is SAM-dependent and was isolated from *S. peucetius*. It transfers a methyl group to the 4-hydroxyl group of carminomycin and its analogues to synthesize the famous anticancer drugs, daunorubicin and its derivatives. The crystal structure of DnrK has been determined by Jansson et al. [76], which provides important information for rational protein engineering of this enzyme. Based on the crystal structure, a mutant DnrK was constructed by replacing its R1 region with the counterpart of a hydroxylase RdmB. The mutant DnrK exhibited both methylase and monooxygenase activities. This chimera was further used to produce a novel anthracycline [77]. Meanwhile, DnrK was employed to produce novel flavonoids depending on the structural similarity of substrates. Surprisingly, DnrK can accept luteolin, luteolin 4′-glucoside, kaempferol, kaempferol-3-O-glucoside and apigenin as the substrate to form their corresponding methylated compounds [78]. These results represented the flexibility of DnrK towards substrates and expanded the spectrum of polyketides. More O-MTs were identified along with new aromatic polyketides discovery. Three O-MTs were deduced to participate in the biosynthesis of four paramagnetoquinones (Pmq A, Pmq B, Pmq C and Pmq D) from *Actinoallomurus* by genome sequencing and amino acids sequence alignment [79]. In order to alleviate the issue of drug-resistant bacteria, a new antibiotic enduracyclinone was discovered from *Nonomuraea sp.* ID40491. This molecule contains methoxy groups and three putative O-MTs were proposed to form the methoxy groups [80]. Although further characterization of these new O-MTs is pending, these studies provided possibilities for expanding the variety of aromatic polyketides by employing new O-MTs.

**Fig. 4:**
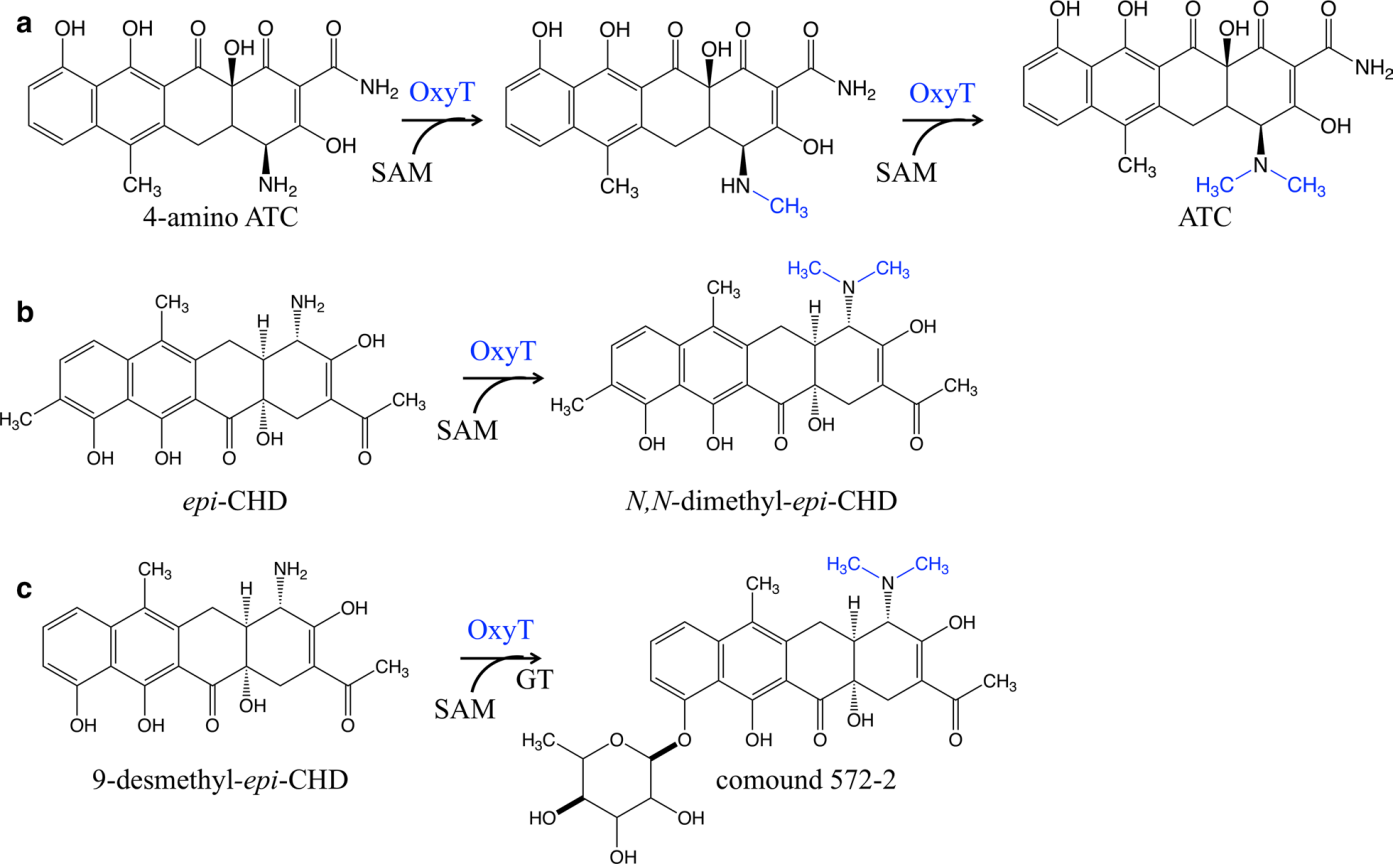
N-methyltransferase OxyT catalyzes mono- and di- methylation. **a** OxyT involved in biosynthesis of ATC. **b** OxyT was employed to catalyze *epi*-CHD generating new compound *N,N*-dimethyl-*epi*-CHD. **c** New compound was produced by employing OxyT and GT. *ATC* anhydrotetracycline, *SAM* S-adenosyl methionine, *OxyT* N-methyltransferase, *CHD* chelocardin, *GT* glycosyltransferase

Compared to O-methylation, N-methylation is less common in the biosynthesis of aromatic polyketides. In 2006, Zhang et al. identified a gene cluster that is responsible for the biosynthesis of oxytetracycline. OxyT was described as a N-MT putatively, because it exhibited high amino acid sequence identity to O-MTs [37]. To further understand the catalytic property of OxyT, 4-amino anhydrotetracycline (ATC) was used as the substrate and incubated with or without purified OxyT in the reaction system. The HPLC/MS result showed that ATC was produced only in the presence of OxyT (Fig. 4a). Remarkably, monomethylated 4-amino ATC was formed (Fig. 4a) as an intermediate when the reaction mixture was stopped at 10 min. Those experiments demonstrated that OxyT is a N-MT that can catalyze both mono- and di-methylating reactions [81] (Fig. 4a). Lukežic et al. designed biosynthetic pathways of 5 novel chelocardin (CHD) analogues in *Amycolatopsis sulphurea* via combining OxyT with an aminotransferase and a glycosyltransferase (Fig. 4b and Fig. 4c) [82]. This research showed the potential of OxyT in the biosynthesis of innovative aromatic polyketides.

In addition to oxidation and methylation, glycosylation also tremendously enhances the diversity of aromatic polyketides. The major glycosylation enzyme is glycosyltransferases (GTs) [83]. UrdGTs were isolated from *S. fradiae* Tü2717 for the biosynthesis of urdamycin. Among the identified UrdGTs encoding genes (urdGT1a, urdGT1b, urdGT1c and urdGT2) by Trefzer et al., UrdGT2 transfers an olivose moiety from dNDP-olivose to rabelomycin and its analogue to generate urdamycinone B and aquayamycin, respectively [84]. This is the first glycosylating step during urdamycin biosynthesis. Then, UrdGT1c and UrdGT1b transfer a rhodinose moiety and an olivose moiety to urdamycinone B sequentially for the generation of compound 100-1 and urdamycin B. UrdGT1c glycosylate aquayamycin to form 100-2, which is the second step involved in the biosynthesis of urdamycin A. Then, UrdGT1c along with UrdGT1b add a rhodinose moiety and an olivose moiety on 100-2 to generate urdamycin A. Four more novel urdamycin derivatives can be synthesized by using various UrdGT1s combinations [85]. This work discovered the function of UrdGT1s and exhibited great potential for expanding the scope of aromatic polyketides by glycosylation. Based on this research, nine novel urdamycins N1–N9 were isolated from *S. diastaticus* subsp. SCSIO GJ056, among which urdamycins N7 and N8 containing (5R, 6R)-angucycline glycosides were discovered for the first time [86]. Saprolmycin A-E are antibiotics isolated from *Streptomyces* sp. TK08046. Three GTs, SprGT1-3, were identified from this producer strain that transfer aculose, rhodinose and olivose moiety to UWM6 (the core structure of saprolmycin), respectively. However, the substrates of these three GTs have not yet to be discovered [87]. As mentioned above, *dnr* gene cluster contributes to biosynthesize daunorubicin. DnrS (encoded by *dnrS*) transfers a daunosamine moiety to rhodomycinne. The latter was converted to daunorubicin catalyzed by DnrP and DnrK [88]. To improve the diversity of daunorubicin family, Chu et al. designed one-pot enzymatic UDP-recycling glycosylation. Two new daunorubicinone glucosides, 7-O-alpha-D glucoside and 7,9-di-O-alpha-D glucoside were generated. These two compounds showed higher thermal stability at 100 °C and tolerance to pH range 4.5–8.5 than daunorubicin [89].

On the contrary of introducing and combining tailoring reactions, inactivation of the corresponding enzymes can also provide some derivatives. The above-mentioned mithramycin can be oxygenated by oxygenase to produce new derivatives. Surprisingly, four new mithramycin analogs could also be obtained by disruption of two genes encoding GTs [90]. Similarly, to explore novel compounds belonging to the trioxacarcin family, Zhang et al. deactivated seven tailoring enzymes including cytochrome P450-dependent oxygenases, MTs and GTs. Eleven trioxacarcin derivatives were generated by the mutants. To further appraise the bioactivity of these analogues, cytotoxicity assays were performed by employing Jurkat cells. The new compound 14a showed similar IC_50_ value with trioxacarcin [42]. These researches provided some potential drug candidates and represent a new direction to improve the diversity of aromatic polyketides. Frigocyclinone was found in *S. griseus* NTK 97 which has antitumor and antibacterial activities. Recently, Mo et al. successfully identified its biosynthetic gene cluster by gene inactivation. An anti-MRSA anthraquinone tetrangomycin was produced by inactivating the gene *frig1* encoding the GT [91]. This study offered another pathway to produce tetrangomycin and its analogs.

## Conclusions

Natural products derived from plants, animals and microorganisms are widely used in food, cosmetic, chemical and pharmaceutical industries. Aromatic polyketides are a special group of natural products synthesized by PKSs and have remarkable bioactivities. The last few decades have witnessed a rapid growth of genetic information acquirement on type II PKSs and an improved understanding of the biological and chemical mechanisms of aromatic polyketide biosynthesis. All the knowledge not only provides exciting opportunities for the rational design of microbial cell factories for high-level biosynthesis of polyketides, but also facilitates the generation of more novel polyketides including “unnatural” derivatives. In order to achieve economically viable production and continually expending the palette of aromatic polyketides, we anticipate the future efforts focused on the following research directions: (1) thorough characterization of catalytic machinery of aromatic polyketides on different levels, such as structural and genetic levels; (2) discovery of more promising aromatic polyketides based on enzyme and gene bioprospecting; (3) production of new aromatic polyketides with improved or novel bioactivities by using combinatorial biosynthesis approach; (4) improvement of catalytic activity of PKSs through protein engineering; (5) exploration and activation of the positive regulatory factor of gene cluster by genetic engineering. We expect that with the continued and rapid development of advanced technologies and tools, more robust and efficient microbial cell factories can be established to achieve economically biosynthesis of type II aromatic polyketides.

## Data Availability

Not applicable.

## Abbreviations

PKSs: Polyketide synthases
AT: Acyltransferase
KS: Ketosynthase
TE: Thioesterase
CoA: Coenzyme A
ACP: Acyl carrier protein
KR: Ketoreductase
DH: Dehydratase
ER: Enoylreductase
iPKSs: Iterative type I PKSs
mPKSs: Modular type I PKSs
ARO: Aromatases
CLF or KS_β_: Chain length factors
CDCHD: 2-carboxamido-2-deacetyl-chelocardin
HLM: Huanglongmycin
2′-PDE: 2′,5′-Oligoadenylate phosphodiesterase
MTs: Methyltransferases
SAM: *S*-adenosyl-l-methionine
GTs: Glycosyltransferases
POK: Polyketomycin
Pmq: Paramagnetoquinones
